# Amphibious Multifunctional Hydrogel Flexible Haptic Sensor with Self-Compensation Mechanism

**DOI:** 10.3390/s24103232

**Published:** 2024-05-19

**Authors:** Zhenhao Sun, Yunjiang Yin, Baoguo Liu, Tao Xue, Qiang Zou

**Affiliations:** 1 School of Microelectronics, Tianjin University, Tianjin 300072, China; sunzhenhao123@tju.edu.cn (Z.S.); 2022232158@tju.edu.cn (Y.Y.); lbg_6227@tju.edu.cn (B.L.); 2 Center of Analysis and Testing Facilities, Tianjin University, Tianjin 300072, China; xuetao@tju.edu.cn; 3 Tianjin International Joint Research Center for Internet of Things, Tianjin 300072, China; 4 Tianjin Key Laboratory of Imaging and Sensing Microelectronic Technology, Tianjin University, Tianjin 300072, China

**Keywords:** wearable, flexible electronics, stability, self-calibration compensation strategy, resistance drift, amphibious environment

## Abstract

In recent years, hydrogel-based wearable flexible electronic devices have attracted much attention. However, hydrogel-based sensors are affected by structural fatigue, material aging, and water absorption and swelling, making stability and accuracy a major challenge. In this study, we present a DN-SPEZ dual-network hydrogel prepared using polyvinyl alcohol (PVA), sodium alginate (SA), ethylene glycol (EG), and ZnSO_4_ and propose a self-calibration compensation strategy. The strategy utilizes a metal salt solution to adjust the carrier concentration of the hydrogel to mitigate the resistance drift phenomenon to improve the stability and accuracy of hydrogel sensors in amphibious scenarios, such as land and water. The ExpGrow model was used to characterize the trend of the ∆*R/R*_0_ dynamic response curves of the hydrogels in the stress tests, and the average deviation of the fitted curves $\bar{\epsilon }$ was calculated to quantify the stability differences of different groups. The results showed that the stability of the uncompensated group was much lower than that of the compensated group utilizing LiCl, NaCl, KCl, MgCl_2_, and AlCl_3_ solutions ($\bar{\epsilon }$ in the uncompensated group in air was 276.158, 1.888, 2.971, 30.586, and 13.561 times higher than that of the compensated group in LiCl, NaCl, KCl, MgCl_2_, and AlCl_3_, respectively; $\bar{\epsilon }$ in the uncompensated group in seawater was 10.287 times, 1.008 times, 1.161 times, 4.986 times, 1.281 times, respectively, higher than that of the compensated group in LiCl, NaCl, KCl, MgCl_2_ and AlCl_3_). In addition, for the ranking of the compensation effect of different compensation solutions, the concentration of the compensation solution and the ionic radius and charge of the cation were found to be important factors in determining the compensation effect. Detection of events in amphibious environments such as swallowing, robotic arm grasping, Morse code, and finger–wrist bending was also performed in this study. This work provides a viable method for stability and accuracy enhancement of dual-network hydrogel sensors with strain and pressure sensing capabilities and offers solutions for sensor applications in both airborne and underwater amphibious environments.

## 1. Introduction

As a material with high water content and adjustable mechanical properties, hydrogel has attracted much attention in recent years, especially showing great potential in the field of pressure sensing. Its application in wearable devices is particularly prominent [1,2,3,4,5,6,7,8,9,10]. Hydrogel-based pressure sensors not only have good flexibility and comfort, but also can realize real-time monitoring of the dynamic pressure of the human body, and thus, they are widely used in medical and health monitoring [11,12,13,14,15,16,17,18,19,20,21,22], smart sports equipment [23,24,25,26,27], and other fields. However, as the application scenarios of hydrogel sensors continue to expand, their stability and accuracy in dynamic environments are challenged due to the fact that gel-based sensors have resistance drift problems due to material degradation and environmental factors [28,29], as well as in underwater environments; hydrogel sensors’ characteristics of absorbing and expanding water [30,31,32] can also affect the accurate monitoring of hydrogel-based sensors. Therefore, further effective solutions are needed to overcome this challenge and enhance the performance of hydrogel sensors used in wearable devices.

To address the background of current operations in performance enhancement of hydrogel sensors, mainly focusing on fabrication processes, material replacement, synthesis methods, optimization of monitoring methods [33,34,35,36,37,38,39,40,41,42,43,44] and other related operations, e.g., Binder et al. [45,46] proposed a force compensation that exploits the additional sensitivity of a doubly sensitive hydrogel by applying force compensation in the opposite stimulation mode, in order to achieve counteracting hydrogel monitoring instability caused by hydrogel swelling. This dual-sensitive hydrogel sensor capable of force compensation was subsequently miniaturized by Binder et al. [47]. In addition, Lee et al. [48] designed a flexible ionogel sensor as a microcone and used a machine learning-based algorithm to assist in monitoring, resulting in a monitoring accuracy of 95.86%. Meanwhile, Wu et al. [49] proposed a way to fabricate a hydrogel sensor suitable for dual sensing of temperature and strain using a salt-permeable strategy, and the sensor exhibited improved mechanical stability, frost resistance, and a wide operating range. Although their approaches improve the performance metrics of hydrogel sensors in different ways, none of them have been able to address the fundamental destabilizing effect on the detection results due to the fact that the polar migration of ions [50,51,52] occurs continuously within the hydrogel and results in resistance drift during monitoring in a conductive working circuit. Thus, this thesis proposes a self-calibration compensation mechanism that provides an idea for the improvement of hydrogel sensor stability and reliability. To address these challenges and improve the performance of hydrogel-based sensors, this study proposes a strategy to introduce compensating ions to mitigate the resistance drift phenomenon. By strategically introducing different ions into the hydrogel matrix, the carrier concentration can be adjusted, and the electrical performance of the sensor can be stabilized. The proposed method of self-calibration compensation is important for improving the stability and reliability of hydrogel-based pressure sensors in air and underwater environments.

In this study, the effectiveness of hydrogels in air and underwater environments is explored, with special attention to the improvement of their stability. And a dual-network hydrogel design combined with a self-calibration compensation strategy is proposed to solve the problem of poor stability of hydrogel-based pressure sensors caused by resistance drift in dynamic environments. Through the characterization and performance evaluation of the system, the effects of the concentration of the compensation solution, the radius of the metal cation in the compensation solution, and the amount of charge carried on the compensation effect of the hydrogel sensor are also analyzed with the aim of demonstrating the importance of our designed hydrogel system and self-calibrated compensation strategy in advancing the field of hydrogel-based pressure sensors.

## 2. Materials and Methods

### 2.1. Materials

Aladdin provided the sodium alginate (SA), while Shanghai National Pharmaceutical Group supplied the polyvinyl alcohol (PVA1750 ± 50, alcoholysis degree ≥ 99%). Tianjin Fuchen Chemical Reagent Co., Ltd., Tianjin, China, provided the ZnSO_4_, and Tianjin Beichen Fangzheng Reagent Factory, Tianjin, China, supplied the ethylene glycol (EG). LiCl was purchased from Shanghai Macklin Biochemical Co., Ltd., Shanghai, China, and NaCl was purchased from Tianjin Zhonglian Chemical Reagent Co., Ltd., Tianjin, China. KCl was procured from Tianjin Huasheng Chemical Reagent Co., Ltd., Tianjin, China, MgCl_2_ from Tianjin Zhiyuan Chemical Reagent Co., Ltd., Tianjin, China, and AlCl_3_ from Tianjin Huasheng Chemical Reagent Co., Ltd., China. Syringes (5 mL capacity, 0.5 × 8 mm needle size) were purchased from Taizhou Ming’an Medical Instrument Co., Ltd., Taizhou, China.

### 2.2. Preparation of DN-SPEZ Hydrogels

**Figure 1:**
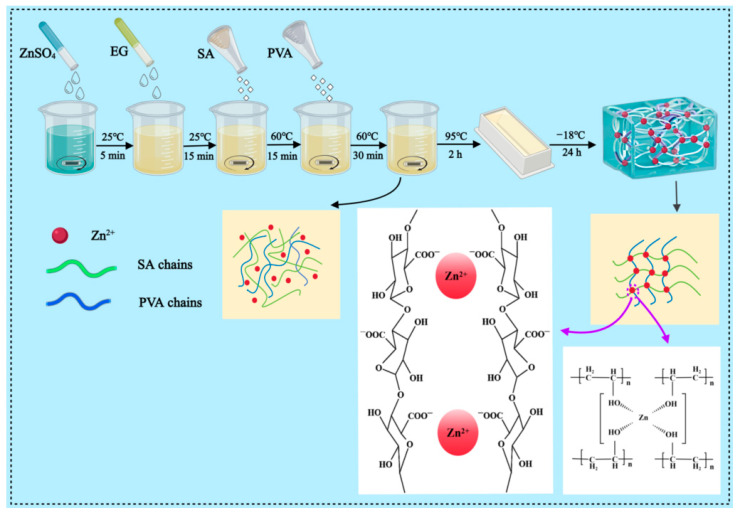
The fabrication process of DN-SPEZ hydrogel and the internal double-network cross-linking form.

First, 8 mL of deionized water and 0.5 g of ZnSO_4_ were stirred uniformly at 200 rad/min for 5 min at room temperature (25 °C) to ensure complete mixing. Next, 2 mL of ethylene glycol (EG) was added in the ratio of 4:1 by volume with deionized water and stirred homogeneously at 200 rad/min for 1 min and then left to stand for 15 min to allow the solution to be thoroughly mixed. Then, 0.3 g of sodium alginate (SA) was stirred at 60 °C for 15 min at 1500 rad/min to dissolve SA and induce a cross-linking reaction. Subsequently, 1.2 g of polyvinyl alcohol (PVA) was added to the mixed solution at 1500 rad/min and stirred at 60 °C for 30 min to induce the swelling of PVA and further formation of the structure of hydrogel. Finally, the mixed solution was stirred at 800 rad/min for 2 h at 95 °C to ensure that the components were fully mixed and formed a stable hydrogel structure. After completion of the above steps, the solution was frozen under ultra-low-temperature conditions (−18 °C) for 24 h and thawed at room temperature (25 °C) for 4 h to form the final DN-SPEZ dual-network hydrogel samples. The fabrication process of the DN-SPEZ hydrogel with the form of internal dual-network cross-linking is shown in Figure 1.

**Figure 2:**
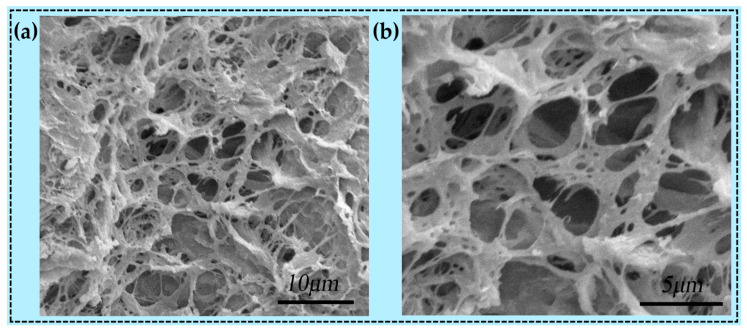
SEM images of DN-SPEZ hydrogels at different magnifications: (**a**) magnification 7000; (**b**) magnification 15,000.

Figure 2 shows the fibrous 3D network structure of the resulting DN-SPEZ hydrogel after lyophilization at a 5 μm scale (magnified 7000 times) and 10 μm scale (magnified 15,000 times). It can be seen that the typical 3D fibrous network structure displayed by DN-SPEZ hydrogels after lyophilization has a thick and rough surface, indicating that the synthesized hydrogels form a stable and physically entangled interpenetrating network structure, which permits the structural stability of the hydrogels as sensors in the event of mechanical deformation [53]. As shown in Figure 2b, the presence of dense pores inside the hydrogel can be observed at the 5 μm scale, which is due to the effect of evaporation of water crystals after lyophilization [54].

### 2.3. Formation of Hydrogels

First, sodium alginate (SA) itself carried a carboxyl group, which interacted electrostatically in the presence of Zn^2+^ to form an ionic bond between Zn^2+^ and the carboxyl group, and this ionic bond aggregated the SA molecule into a three-dimensional network structure by cross-linking [55]. Secondly, the divalent cation Zn^2+^ provided four empty orbitals through sp^3^ hybridization [56], while the hydroxyl group carried by polyvinyl alcohol (PVA) provided a lone pair of electrons, which resulted in the formation of a ligand bond between Zn^2+^ and PVA, and this ligand bond cross-linking led to the formation of a three-dimensional spatial mesh structure of PVA. At the same time, a large number of water molecules and ethylene glycol (EG) molecules were wrapped between the PVA meshes, which together formed a hydrogel structure through hydrogen bonding.

On this basis, Zn^2+^ was ionically cross-linked with SA and coordinated with PVA, respectively, and physical entanglement between the two networks occurred, forming an interpenetrating dual-network cross-linked polymer hydrogel. This dual-network structure retains the reticular structure formed by ionic cross-linking between SA and Zn^2+^ and also includes the three-dimensional spatial reticular structure formed by coordination cross-linking between PVA and Zn^2+^. This interpenetrating dual-network structure endows the hydrogel with excellent mechanical properties and stability. Meanwhile, due to the large number of hydroxyl groups on the surface of EG, hydrogen bonds were formed with water molecules, which enhanced the mechanical properties of the hydrogel and improved the frost resistance and moisturizing properties [57].

In addition, the hydrogen bonding between the molecular chains of PVA and EG strengthened the internal network of the hydrogel, while the aqueous solution of PVA further enhanced the stability of the hydrogel through the physical cross-linking of hydrogen bonding with the crystals during the freeze–thaw process. Finally, ZnSO_4_ formed conductive ion channels in the co-solvent mixture of EG and water and was isolated by EG. When ZnSO_4_ was added to the hydrogel, in addition to Zn^2+^, which was involved in the synthesis of the long-chain structure of the hydrogel, the ionization of the remaining ZnSO_4_ was able to generate a sufficient number of free-moving ions, which contributed to the improvement of the electrical conductivity of the hydrogel.

### 2.4. Implementation of a Self-Calibrating Compensation Strategy

In order to solve the problem of resistance drift in hydrogel sensors, this paper adopts an ion compensation strategy to regulate the carrier concentration inside the hydrogel. During the sensor operation, when the hydrogel undergoes carrier polarity migration resulting in an increase in resistance, the ions in the compensation solution added to the interior will continuously diffuse into the hydrogel with osmosis [58] to compensate for the hydrogel carriers. Through this strategy, the resistance drift trend of the hydrogel can be significantly slowed down or even stabilized, which improves the stability and accuracy of the hydrogel sensor in detecting stress and strain and achieves the effect of self-calibration.

**Figure 3:**
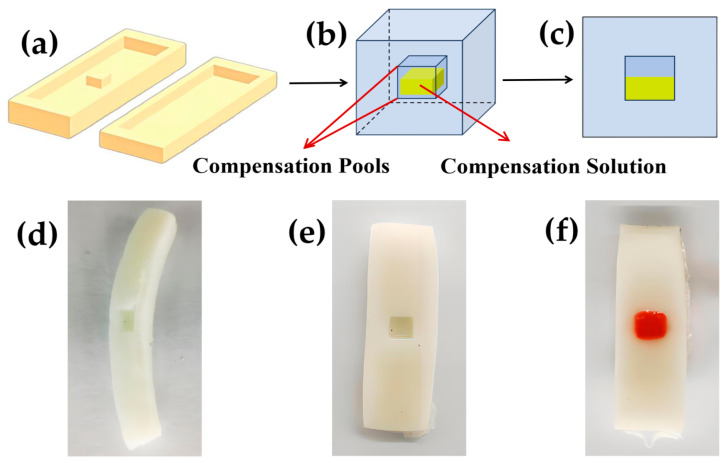
The implementation scheme of the self-delivery quasi-compensation mechanism: (**a**) Hydrogel freeze–thaw molding mold; (**b**) perspective view of a hydrogel injected with compensation solution; (**c**) schematic view of hydrogel injected with compensation solution; (**d**) hydrogel cross-section; (**e**) hydrogel profile; (**f**) profile after hydrogel compensation.

The specific realization of the process is shown in Figure 3. After the hydrogel preparation, the mixed solution is poured into the molds (Figure 3a), and a regular slot structure is designed inside the molds. The existence of this groove structure makes the hydrogel freeze–thaw with the internal formation of an empty groove, and the size of the empty groove is 5 mm × 5 mm × 2 mm. Then, a syringe (5 mL capacity, 0.5 × 38 mm needle specifications) is used for the injection of a metal cation salt solution; through the absorption of hydrogel, ions in the interior of the hydrogel participate in ionization to achieve the effect of the compensation of the resistance of the hydrogel drift and thus achieve the self-calibration. Then, the purpose of self-calibration is achieved. The hydrogel side section structure (Figure 3d), profile structure (Figure 3e), and hydrogel profile (Figure 3f) state of the hydrogel injected with ion compensation solution are also shown.

## 3. Results

### 3.1. Mechanical Properties of DN-SPEZ Hydrogels

**Figure 4:**
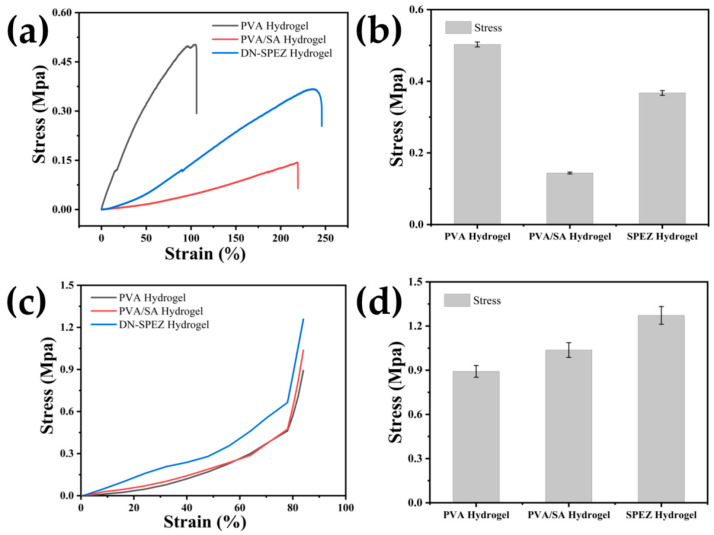
DN-SPEZ hydrogel mechanical properties: (**a**) tensile curves of hydrogels with different formulations; (**b**) corresponding maximum tensile stress; (**c**) compression curves of hydrogels with different formulations; (**d**) corresponding maximum compressive stress.

Compared with PVA hydrogels and PVA/SA hydrogels, DN-SPEZ hydrogels exhibited superior mechanical properties, including compression and tensile properties (see Figure 4a). We believe that this can be explained from three aspects: (1) firstly, the dual-network structure of the hydrogel and the hydrogen bonding formed between the EG surface carrying a large number of hydrophilic groups and water molecules enhanced the mechanical properties of the hydrogel; (2) the coordination bond formed by PVA through the coordination with Zn^2+^ can effectively extend the length of the polymer chain, which allows the tension to be uniformly transferred to the extended polymer chain, giving the DN-SPEZ hydrogels higher tensile and ductile properties; (3) since EG acts as a lubricant after its introduction into the hydrogel, it makes the polymer chains slide more easily and reduces the friction between the polymer chains [59].

Tensile and compression tests were conducted on PVA hydrogels, PVA/SA hydrogels, and DN-SPEZ hydrogels. The typical tensile curves of the different hydrogel formulations are shown in Figure 4a. The maximum tensile change for PVA hydrogel was only 106.2%, while for PVA/SA hydrogel, it was 219.3%. In contrast, the maximum tensile change for DN-SPEZ hydrogel was 245.9%, which is 2.32 times higher than that of PVA hydrogel and PVA/SA hydrogel. The results showed that the dual-network hydrogel was 2.32 times more stable and 1.12 times more yieldable than PVA/SA hydrogel. The study found that the dual-network structure of the hydrogel enhances its stability and increases the yield limit of the material. Additionally, the dual-network hydrogel has a larger tensile range after the introduction of EG, making DN-SPEZ better suited for practical applications that require a wider range of strain monitoring. Figure 4b illustrates the maximum stresses experienced by the PVA hydrogel, PVA/SA hydrogel, and DN-SPEZ hydrogel during the tensile process, which were 0.503 MPa, 0.144 MPa, and 0.367 MPa, respectively. Compared to the maximum tensile change of DN hydrogel (~140%) mentioned by Zeng et al. [60], the maximum tensile change of DN-SPEZ hydrogel in this paper (245.9%) presents an advantage, which is also greater than the mechanical criterion of the maximum strain of hydrogel sensors for monitoring human body movements (~55%) [61].

Additionally, we conducted a compression test to examine the impact of EG on the dual-network hydrogel. As shown in Figure 4c,d, the maximum compressive stress of the PVA hydrogel at 90% strain was 0.89 MPa, while for the PVA/SA hydrogel, it was 1.04 MPa, and for the DN-SPEZ hydrogel, it was 1.27 MPa. Zeng et al. [60] mentioned that the maximum compressive stress of DN hydrogel in reaching 90% deformation is 321.1 kpa, which is much lower than that of DN-SPEZ (1.27 MPa) in terms of compressive capacity. This suggests that the DN-SPEZ hydrogel, prepared in this study, has superior compressive properties and can withstand greater pressure without damage or deformation. Compared to the other two hydrogels, the DN-SPEZ hydrogel exhibits higher strength and stiffness, making it suitable for underwater applications that require superior compressive properties.

### 3.2. Physical Properties of DN-SPEZ Hydrogels

**Figure 5:**
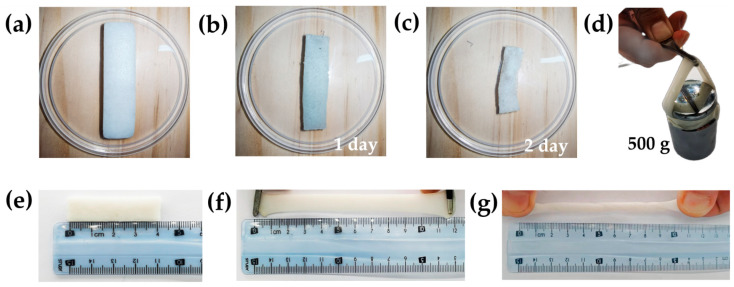
(**a**–**c**) DN-SPEZ hydrogel states at different time; (**d**) weightlifting; (**e**,**f**) stretching; (**g**) DN-SPEZ hydrogel post-twist stretching.

The physical properties of hydrogels are crucial for their practical applications due to the strict requirements of their mechanical properties in the environment they are exposed to. Figure 5a–c display the sample states of the DN-SPEZ hydrogel used in this paper for different time periods. Figure 5d demonstrates that the hydrogel can withstand a weight of 500 g. Figure 5e,f show the length of the DN-SPEZ hydrogel before and after stretching, respectively. The initial length was 42 mm, and the post-stretching length was 121 mm, which is approximately 2.88 times the initial length. Additionally, even after the hydrogel is twisted by 1080°, its length can still be stretched to 110 mm (Figure 5g), which is approximately 2.62 times the initial length. The results suggest that DN-SPEZ hydrogels exhibit exceptional toughness and ductility.

**Figure 6:**
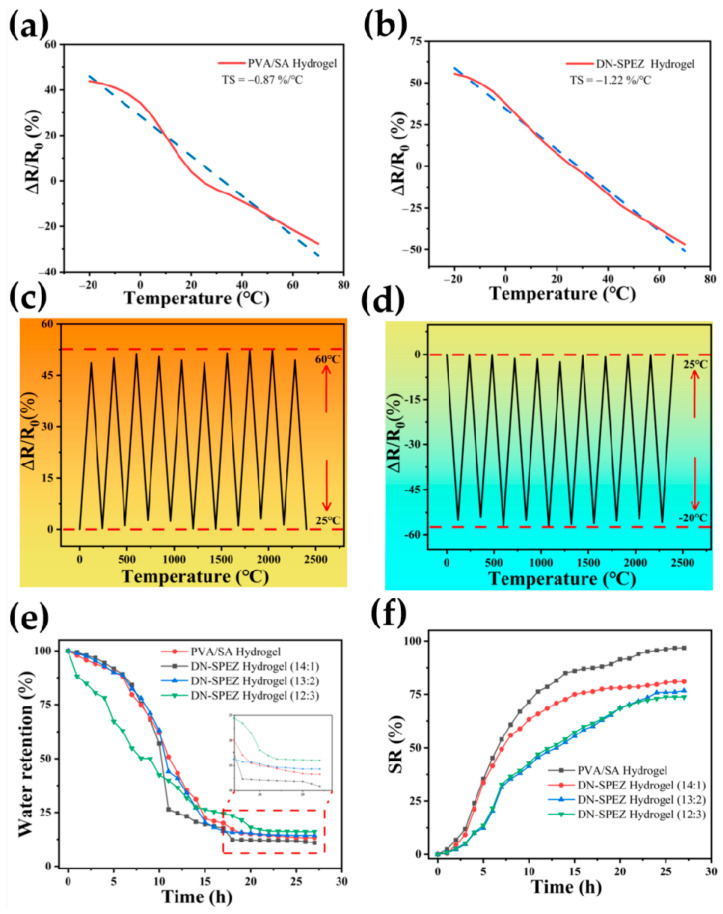
(**a**) PVA/SA hydrogel temperature-sensitive properties; (**b**) DN-SPEZ hydrogel temperature-sensitive properties; (**c**) stability of DN-SPEZ hydrogel in the range of 25 °C–60 °C for 10 cycles; (**d**) stability of DN-SPEZ hydrogel in the range of −20 °C to 25 °C for 10 cycles; (**e**) water retention of PVA/SA hydrogel vs. DN-SPEZ hydrogel with different EG contents; (**f**) dissolution rate of PVA/SA hydrogels vs. DN-SPEZ hydrogels with different EG contents.

With the introduction of EG, the DN-SPEZ hydrogel possesses excellent low-temperature tolerance, which offers the possibility of its use as a temperature sensor. Figure 6a,b demonstrate the temperature-sensitive properties of DN-SPEZ hydrogel and PVA/SA hydrogel without added EG in the temperature range from −20 °C to 60 °C. The hydrogel is characterized by a negative temperature coefficient (NTC), which is the same as the temperature sensitivity. As the temperature increases, the resistance of the hydrogel decreases and exhibits a negative temperature coefficient (NTC) property, which also visualizes the temperature detection capability of the hydrogel sensor from the perspective of temperature sensitivity. Temperature sensitivity (*TS*) is defined as follows [Equation (1)] [62]:

(1) $$TS=\frac{R-R_{0}}{R_{0}\times ∆T}$$

The formula defines *R*_0_ as the initial resistance, *R* as the resistance at a specific temperature, and Δ*T* as the difference between the current temperature and the initial temperature. The unit of temperature sensitivity is expressed as %/°C. The temperature sensitivity characteristic curves of Figure 6a,b were linearly fitted using origin software, and the slopes of the fitted curves showed that the temperature sensitivity of the DN-SPEZ hydrogel sensor with EG addition was −1.22%/°C in the temperature range of −20–60 °C. In comparison, the temperature sensitivity of the hydrogel without added EG was only −0.87%/°C. Meanwhile, the linearity of the temperature sensitivity characteristic curve of DN-SPEZ hydrogel with EG addition was more obvious compared with that of the hydrogel without added EG. Xu et al. [63] synthesized and assembled a Li^+^/agar/pHEAA DN dual-network hydrogel sensor for stress–strain monitoring of human body movement, and it had good nonlinear temperature-sensitive characteristics only in the interval of 30–60 °C, whereas DN-SPEZ presents not only a better temperature range, but also a better linearity in the temperature sensitivity curve.

Figure 6c,d demonstrate the temperature sensitivity reproducibility of DN-SPEZ hydrogel at temperatures ranging from 25 °C to 60 °C and from −20 °C to 25 °C, respectively, although it was different from that of the hydrogel of Zou et al. [62], who proposed an SNPG multimodal hydrogel sensor, for which the degree of temperature response was relatively low (∆*R/R*_0_ of SNPG is about 143% at −20 °C and 68% at 55 °C); the ∆*R/R*_0_ of the DN-SPEZ hydrogel is 52.34% at −20 °C and 57.26% at 60 °C, but it still demonstrates a good temperature-sensing repeatability in temperature sensing. The wide temperature measurement range and stability further endow the hydrogel sensor with the potential to become a wearable device, e.g., for extreme ambient temperature detection or for helping the human body to collect elements of environmental information.

Figure 6e shows the water retention ratio of the hydrogel before and after the introduction of different amounts of EG in a room temperature environment (25 °C, 50% RH), which is described as the water retention behavior of the hydrogel. The ratio in the legend is the volume ratio of deionized water to EG. The hydrogels were weighed and placed at room temperature and weighed after water loss at one-hour intervals over a period of 27 h. The water retention (WR) of the hydrogel was defined as follows [Equation (2)] [64]:

(2) $$Water\;rentention=\frac{W_{S}}{W_{0}}$$

where *W*_0_ is the initial weight of the hydrogel and *W_S_* is the weight measured at the current moment after the hydrogel lost water. At the 27th hour, the dissolution rate of the PVA/SA hydrogel was 10.90%, whereas the water retention levels of the DN-SPEZ hydrogels with the volume ratios of deionized water to EG of 14:1, 13:2, and 12:3 were 13.31%, 14.33%, and 16.06%, which were only 1.22, 1.31, and 1.47 times higher than those of the former, respectively. With the increasing content of introduced EG, it can be seen that the water retention performance of hydrogel at room temperature is gradually improved, and EG can confer good water retention ability to hydrogel [59]. In addition, Tang et al. [65] have also proposed an approach to improve the water retention capacity of DN hydrogels by increasing the concentration of CaCl_2_ in the hydrogel. Due to the strong hygroscopicity of CaCl_2_, DN hydrogels containing 30 wt.% of CaCl_2_ can capture water molecules from the atmosphere. Based on the strong hygroscopicity of CaCl_2_, the dehydrated DN hydrogel can regenerate itself by spontaneously capturing water molecules from the surrounding environment.

Figure 6f demonstrates the water absorption and swelling characteristics of the hydrogel before and after the introduction of different amounts of EG, which is described as the swelling behavior of the hydrogel. The ratio in the legend indicates the volume ratio of deionized water to EG. The hydrogels were weighed and immersed in deionized water at room temperature and were chosen to be weighed after water absorption and swelling at one-hour intervals over a period of 27 h. The swelling rate (*SR*) of the hydrogel was defined as follows [Equation (3)] [66]:

(3) $$SR=\frac{W_{S}-W_{0}}{W_{0}}$$

At the 27th hour, the *SR* of the PVA/SA hydrogel was 96.82%, while the swelling rates of the DN-SPEZ hydrogel with the volume ratios of deionized water to EG of 14:1, 13:2, and 12:3 were 80.96%, 76.68%, and 73.75%, respectively, which were only 0.836, 0.792, and 0.762 times higher than those of the former, respectively. Compared to the Gel@BC bilayer hydrogel proposed by Hou et al. [67], the swelling rate of the DN-SPEZ hydrogel with a volume ratio of deionized water to EG of 12:3 (36.57%) was much lower than that of the Gel@BC bilayer hydrogel at the optimum ratio (~75%) at the 8th hour of the experiment. We believe that the results of the water retention and swelling tests can be explained by two main mechanisms. First, the incorporation of EG molecules, through the formation of hydrogen bonding interactions with water molecules, will form a protective layer that slows down the rate of penetration of water molecules into the internal structure of the hydrogel, thus limiting the free diffusion of water molecules in the hydrogel. Second, the presence of EG molecules hinders the free diffusion of water molecules in the hydrogel, further reducing the penetration rate of water molecules. The combination of these two effects led to a certain reduction in the swelling rate of the hydrogels with the addition of EG. By adding EG, it was found that the hydrogel swelling rate could be effectively slowed down, making our sensor less susceptible to dimensional changes due to water-absorbing swelling under operating conditions, in order to improve the stability and reliability of the monitoring of DN-SPEZ hydrogel sensors, which is crucial for sensors that need to work stably for long periods of time and be used under complex environmental conditions. Therefore, by introducing EG into the hydrogel composition to enhance the anti-swelling properties of the hydrogel, the stability of the sensor during the monitoring process can be enhanced, which can better meet the practical needs of this sensor in many amphibious scenarios.

### 3.3. Pressure Sensitivity and Strain Sensitivity

**Figure 7:**
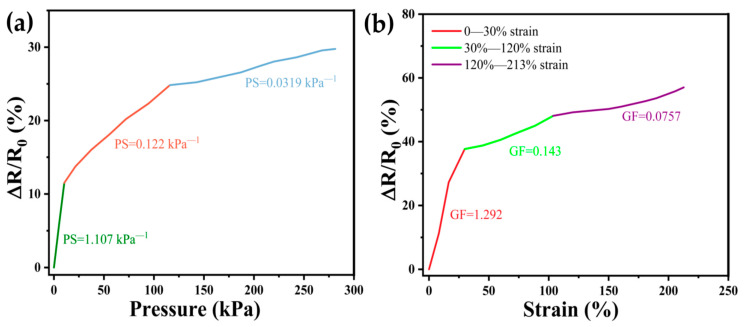
Pressure sensitivity and strain sensitivity: (**a**) relationship between resistance change and pressure; (**b**) relationship between resistance changes and tensile stresses.

Combining the mechanical properties and electrical conductivity of the hydrogels, we comprehensively analyzed and studied the pressure sensitivity and tensile sensitivity of DN-SPEZ hydrogels. As shown in Figure 7a, the relative resistance change of DN-SPEZ hydrogel changed significantly when it was squeezed, and since the trend of resistance change of DN-SPEZ under different pressure strains was not consistent, the pressure sensitivity (*PS*) of DN-SPEZ hydrogel was calculated by ∆*R/R*_0_ with the pressure (*P*) applied to the hydrogel according to the following equation [Equation (4)] [62]:

(4) $$PS=\frac{R-R_{0}}{R_{0}\times ∆P}$$

A change in pressure sensitivity (*PS*) was observed. The *PS* was 1.107 kPa^−1^ at an applied pressure of 0–10.4 kPa, 0.122 kPa^−1^ at 10.4–116 kPa, and 0.0319 kPa^−1^ at 116–281.3 kPa. *PS* changes in hydrogels are mainly influenced by their internal structure, which consists of polymer chains and adsorbed water molecules. In the low-pressure state, the distance between the polymer chains and water molecules within the hydrogel is large, making the polymer chains more flexible and the water molecules free to move. When pressure is applied externally, it leads to an increase in the carrier migration speed inside the hydrogel, while the network structure inside the neighboring hydrogel generates more conductive pathways due to the initial squeezing contact [68], which results in a larger change in the electrical signal and makes the sensor highly sensitive. However, with a further increase in pressure, the distance between the polymer chains and water molecules within the hydrogel gradually decreases, resulting in the double network structure of the hydrogel interpenetration becoming tighter, limiting the movement of carriers. This structural change reduces the sensitivity of the hydrogel to external pressure because the reduced spacing of the internal network decreases the space for the carriers to move, resulting in a reduced change in the electrical signal when the hydrogel is subjected to the same pressure change, which reduces the sensitivity of the sensor. The DN hydrogel mentioned by Zeng et al. [60] shows the same segmentation of the pressure sensitivity in the 0–1 kPa and 1–3.5 kPa intervals despite the linearity of the pressure sensitivities, compared to the DN-SPEZ hydrogel mentioned in this paper. The gel is larger in the pressure range (0–281.3 kPa) compared to the DN-SPEZ hydrogel mentioned in this paper.

In addition to the pressure sensitivity performance, we also investigated the tensile strain sensitivity of DN-SPEZ hydrogels. As shown in Figure 7b, the relative resistance change of the hydrogel, ∆*R/R*_0_, was significantly correlated with the tensile strain (Strain). When the strain reaches 213%, the relative change in resistance ∆*R/R*_0_ reaches 57%. We found that the GF varied continuously in different tensile strain ranges, and the tensile range Δ*R/R*_0_ tended to vary linearly in the 30–120%, 30–120%, and 120–213% segments; the linear fitting function of the origin software was utilized to calculate the strain sensitivity (GF). In the 0–30% tensile strain range, the GF was 1.292; in the 30–120% strain range, the GF was 0.143; and in the 120–213% strain range, the GF was 0.0757. In smaller tensile ranges, the internal structure of the hydrogel changed more significantly, and the resistance change was more sensitive. With the increase in stretching range, the internal structure change of hydrogel is weakened, and the degree of resistance change is reduced accordingly, resulting in a decrease in GF. We believe that this phenomenon is due to the deformation of the internal structure of the hydrogel after stretching; the ion channel shrinks and narrows, and the polar migration path of ions becomes longer, but the degree of lengthening gradually decreases, and the rate of resistance growth slows down [69]. Compared to the DN hydrogel mentioned by Zeng et al. [60], the DN-SPEZ hydrogel has a larger range of tensile strain. The above results highlight the advantages of DN-SPEZ hydrogel in terms of pressure and strain sensitivity, providing practical value and significance for its application in the field of stress–strain sensors. This DN-SPEZ hydrogel with good sensitivity and tunable performance can be widely used in the fields of medical treatment, health monitoring, and human–computer interaction.

### 3.4. Compensation Effects of Performing Self-Calibrating Compensation Strategies Using Different Solutions

We chose LiCl, NaCl, KCl, MgCl_2_, and AlCl_3_ solutions as the compensation solutions for hydrogels to perform self-calibration for the study. On the one hand, lithium, sodium, and potassium, as elements of the same group, have different degrees of influence on the conductivity of hydrogels due to the differences in their electron arrangement and ionic radii in the ionic state. On the other hand, sodium, magnesium, and aluminum, as elements of the same cycle, also exhibit differences in electrical properties due to differences in atomic nuclear charges, which in turn affect their migration states in the hydrogel, despite their similarities in electron arrangement. Therefore, the rationality of choosing lithium, sodium, potassium, magnesium, and aluminum for carrier compensation testing lies in the ability to utilize their similarities and differences to comprehensively assess the effects of different compensation solutions on hydrogel properties.

**Figure 8:**
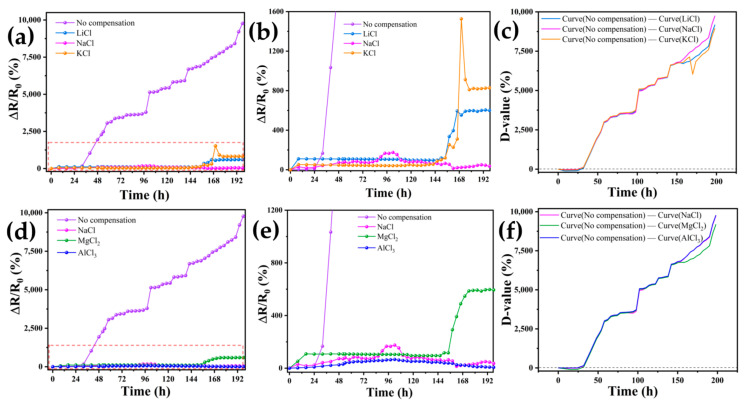
(**a**) Resistance drift of hydrogel in energized circuit after compensation of uncompensated group, LiCl, NaCl, and KCl; (**b**) partial enlargement of Figure 8a; (**c**) trend of the difference between the hydrogel resistance drift curves of the uncompensated group and the LiCl, NaCl, and KCl compensation; (**d**) resistance drift of hydrogel in energized circuit after compensation of uncompensated group, NaCl, MgCl_2_, and AlCl_3_; (**e**) partial enlargement of Figure 8d; (**f**) trend of the difference between the hydrogel resistance drift curves of the uncompensated group and the NaCl, MgCl_2_, and AlCl_3_ compensation.

As shown in Figure 8, hydrogels made from the same batch were taken and injected with 0.05 mL of a 1 mol/L solution of LiCl, NaCl, KCl, MgCl_2_, or AlCl_3_. They were then connected to the test circuit at room temperature to start an 8-day resistance test to verify the effectiveness of the ion compensation measures described in this paper. A control group of uncompensated hydrogels was also chosen for the experiment. Figure 8a shows the changes in resistance of LiCl, NaCl, and KCl ion-compensated hydrogels over 192 h. The uncompensated control group had a continuously increasing resistance, with a ∆*R/R*_0_ of 9772.35% by the 192nd hour of the experiment. In contrast, the ion-compensated hydrogels had a slower rising trend, with the hydrogel compensated with KCl solution showing the slowest rising trend. The resistance of the hydrogel compensated with KCl reached 9772.35% during the test. During the test, the maximum value of ∆*R/R*_0_ for the hydrogel compensated with KCl solution was only 1526.67%, while the control group had a ∆*R/R*_0_ 6.4 times higher than that of the hydrogel compensated with KCl solution. To clearly show the compensation effect of various solutions, Figure 8b presents a local magnification of Figure 8a. The LiCl-compensated hydrogel ∆R/R_0_ exhibited a normal upward trend in the first 10 h of the experiment, followed by a slowdown in growth, and finally a rapid upward trend. On the other hand, the NaCl-compensated hydrogel ∆*R/R*_0_ initially showed slow growth, followed by a downward trend in growth as shown in Figure 8a,b. Figure 8c illustrates the relationship between the hydrogel drift curves of the uncompensated experimental group and the drift curves of LiCl, NaCl, and KCl after compensation at the 192nd hour of the experiment. The reduction reached 9171.6% after LiCl compensation, 9736.1% after NaCl compensation, and 8943.5% after KCl compensation, compared to the ∆*R/R*_0_ of the uncompensated hydrogel.

Figure 8d shows the resistance change of hydrogels with ionic compensation by NaCl, MgCl_2_, and AlCl_3_. The hydrogel compensated with MgCl_2_ solution had a maximum ∆*R/R*_0_ value of only 597.93% during the test, while the maximum resistance change of the control group was 16.3 times that of the hydrogel compensated with MgCl_2_ solution. The solutions of NaCl, MgCl_2_, and AlCl_3_ all slowed down the growth of the hydrogel resistance trend after compensation. Figure 8e shows a localized enlargement of Figure 8d. Figure 8f demonstrates the relationship between the difference in hydrogel drift curves of the uncompensated experimental group and the drift curves of LiCl, NaCl, and KCl after compensation at the 192nd hour of the experiment. The reduction reached 9178.1% after MgCl_2_ compensation and 9765.5% after AlCl_3_ compensation. Therefore, we believe that the compensation solution of the hydrogel in this paper is due to osmotic pressure and water absorption. The ions enter the hydrogel and increase the concentration of carriers, which alleviates the effect of hydrogel resistance drift.

Although the hydrogel sensor still suffers from a continuous increase in resistance due to carrier polarity migration during operation, the use of the compensation solution significantly mitigates this trend, allowing the compensated hydrogel to exhibit better stability and accuracy. Therefore, this self-calibrating compensation strategy provides practical value for the application of hydrogel sensors, which can ensure the accuracy and stability of the monitoring data, thus improving the performance of the sensors and making them more reliable in practical applications.

### 3.5. Application Effect of DN-SPEZ Hydrogel Self-Calibrating Compensation Sensor

**Figure 9:**
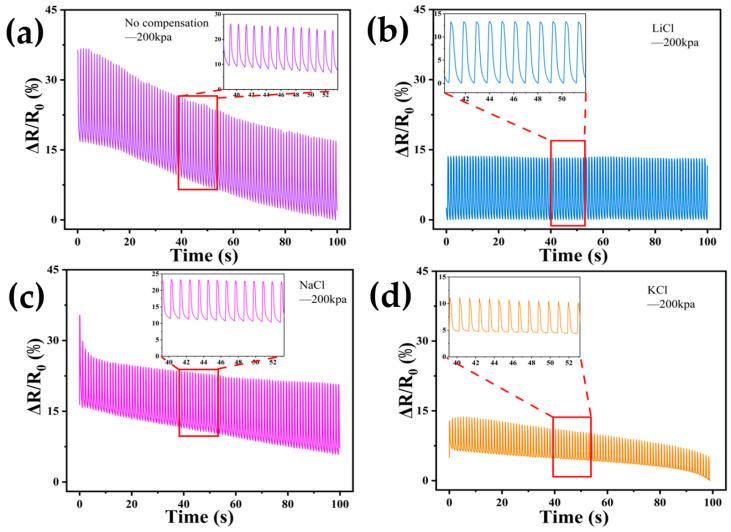
Resistance change in cycling test at 200 kPa pressure: (**a**) uncompensated experimental; (**b**) LiCl compensation; (**c**) NaCl compensation; (**d**) KCl compensation.

In order to investigate the characteristics of DN-SPEZ hydrogels after the self-calibration of pressure sensors using different compensation solutions, we still chose LiCl, NaCl, KCl, MgCl_2_, and AlCl_3_ solutions for the self-calibration compensation and performed pressure tests on the compensated hydrogels using a universal test bench with a test pressure of 200 kPa. Figure 9a,d demonstrate the pressure fatigue test of DN-SPEZ hydrogel after ionic compensation using LiCl, NaCl, and KCl with the same main group metal cation salt solution and the uncompensated DN-SPEZ hydrogel. LiCl has a better compensating effect, and we believe that Li^+^, Na^+^, and K^+^ are all monovalent cations carrying the same charge in the atomic structure, while Li^+^ has a smaller ionic radius (0.76 Å) [70], so the charge density of Li^+^ is larger, giving Li^+^ hydrogel internal network channels higher ion mobility, so in the hydrogel sensor conductive pathway, Li^+^ can be more effective in supplementing the carriers in the hydrogel, slowing down the phenomenon of ion migration within the hydrogel, so as to improve the resistance stability of the hydrogel, which will make the results of the pressure fatigue test more stable and reduce the drift phenomenon. The compensation effect of NaCl and KCl is the next best; due to the relatively large ionic radii (Na^+^: 1.02 Å, K^+^: 1.38 Å) [70], the corresponding charge concentration is lower, the ionic mobility is lower compared to Li^+^, and the efficiency of compensating for the carriers in the hydrogel is also relatively poor, so the resistance of the hydrogel has a poorer compensation effect.

In summary, the self-calibration compensation of hydrogels using different ion solutions is an effective strategy to significantly reduce the hydrogel resistance drift phenomenon. Although the introduction of some ions may lead to a reduction in the pressure sensitivity of the hydrogel, stability is crucial in hydrogel pressure sensing applications. Therefore, even though the pressure sensitivity is sacrificed to some extent, the stability of the hydrogel resistance is ensured by the compensation mechanism, which will be of positive significance in realizing the reliability and long-term stability of hydrogels in the field of pressure sensing.

**Figure 10:**
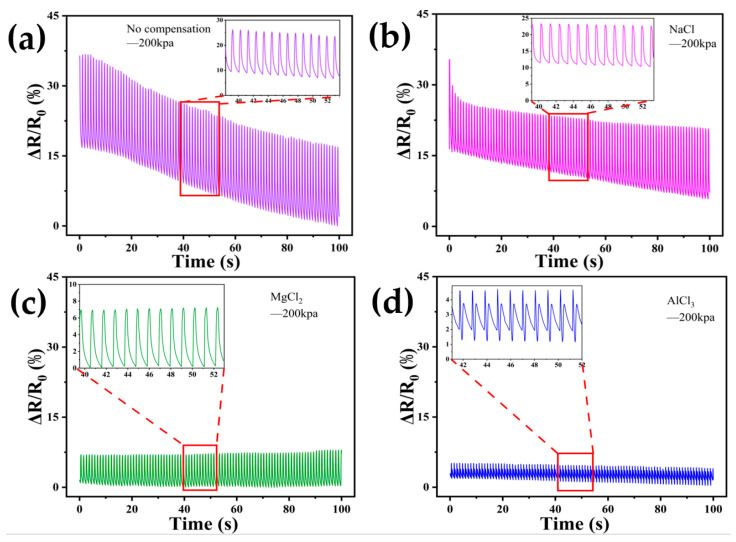
Resistance change in cycling test at 200 kPa pressure: (**a**) uncompensated experimental; (**b**) NaCl compensation; (**c**) MgCl_2_ compensation; (**d**) AlCl_3_ compensation.

As shown in Figure 10a–d, the pressure fatigue tests of DN-SPEZ hydrogels after ionic compensation using NaCl, MgCl_2_, and AlCl_3_ for the same cycle of metal cation salt solutions and uncompensated DN-SPEZ hydrogels are demonstrated. We observed that the MgCl_2_ and AlCl_3_ compensation showed a more stable trend with less drift in the pressure fatigue test of the hydrogels, while the NaCl compensation performed poorly. The Mg^2+^ and Al^3+^, while possessing smaller ionic radii (Mg^2+^: 0.72 Å, Al^3+^: 0.535 Å) [70], carry larger charges, allowing them to have higher charge densities as well as higher ion mobility within the hydrogel, thus providing a stable source of carriers more efficiently during the compensation process, which is conducive to maintaining the conductivity of the hydrogel and thus reducing the drift phenomenon in the test. In contrast, Na^+^ has a lower charge density and lower ion mobility, making its compensation performance worse compared to Mg^2+^ and Al^3+^, but it still shows a compensation effect compared to the uncompensated control group.

**Figure 11:**
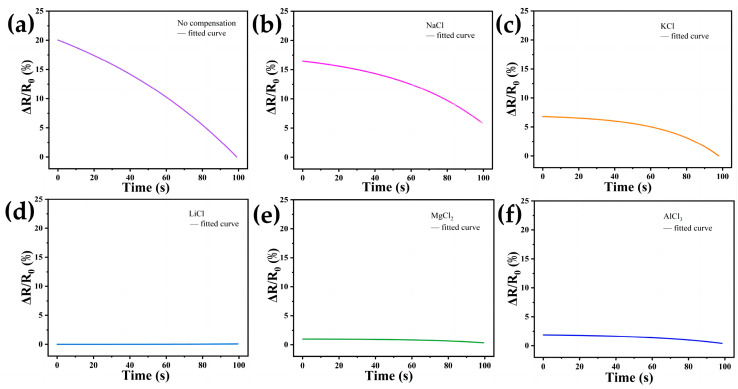
Stability fit curve of DN-SPEZ hydrogel for pressure testing in air: (**a**) uncompensated experimental; (**b**) LiCl compensation; (**c**) NaCl compensation; (**d**) KCl compensation; (**e**) MgCl_2_ compensation; (**f**) AlCl_3_ compensation.

In order to be able to quantify the effect of the hydrogel after performing the self-calibration compensation strategy, inspired by the way D. Lewis [71], Bandari [72], and Adam [73] dealt with the processing of the nonlinear response data handling, we decided to use the nonlinear curve-fitting function of the origin software to fit the self-calibration compensation of the LiCl, NaCl, KCl, MgCl_2_, and AlCl_3_ solutions. For the pressure response curves, as shown in Figure 11, we used the ExpGrow model with a better fitting effect, as shown in the following equation [Equation (5)]:

(5) $$f(x)={f(x}_{0})+A_{1}\times e^{\frac{x-x_{0}}{t_{1}}}$$

where f(x) describes the ∆*R/R*_0_ of the fitted curve for the autocovariance time x, *A*_1_ is the amplitude parameter of the exponential term, $x_{0}$ is the translation parameter of the exponential function, $f(x_{0})$ is the initial offset when $x=x_{0}$, and *t*_1_ is the decay/growth rate parameter of this exponential function, which controls the rate of change of this function. To clarify the numerical difference between the fitted value of the curve and the actual value of the offset, the following equations were used [Equations (6) and (7)]:

(6) $$\epsilon_{i}=\left|y_{0}-{f(x}_{i})\right|$$

(7) $$\bar{\epsilon }=\frac{1}{n}\sum_{i=1}^{n}\epsilon_{i}$$

where $y_{0}$ represents the initial value of ∆*R/R*_0_ in the actual test curve, $x_{0}$ represents the ∆*R/R*_0_ value of the fitted curve at that time, $\epsilon_{i}$ represents the absolute deviation between the fitted value and the actual initial value, and $\bar{\epsilon }$ represents the average deviation value. The size of the $\bar{\epsilon }$ value corresponding to the different experimental groups represents the stability of the sensor in the experimental process. The larger the value, the greater the offset of the fitted curve, i.e., the more unstable; conversely, the smaller the value, the better the stability of the sensor during use. The average deviation $\bar{\epsilon }$ for the uncompensated group tested in air was calculated to be 0.02004269. For the LiCl-compensated group, it was 0.00007261; for NaCl-compensated, it was 0.01061724; for KCl, it was 0.00675606; for MgCl_2_, it was 0.00065474; and for AlCl_3_, it was 0.00147718. The average deviation of the resistance drift of the uncompensated group in air is 276.158, 1.888, 2.971, 30.586, and 13.561 times that of the LiCl, NaCl, KCl, MgCl_2_, and AlCl_3_ compensation groups, respectively, and the larger the multiplier is, the better the compensation effect is. The results obtained indicate that the stability of the sensors in exposed air applications is improved compared to the uncompensated sensors after self-calibration compensation using LiCl, NaCl, KCl, MgCl_2_, and AlCl_3_ solutions.

**Figure 12:**
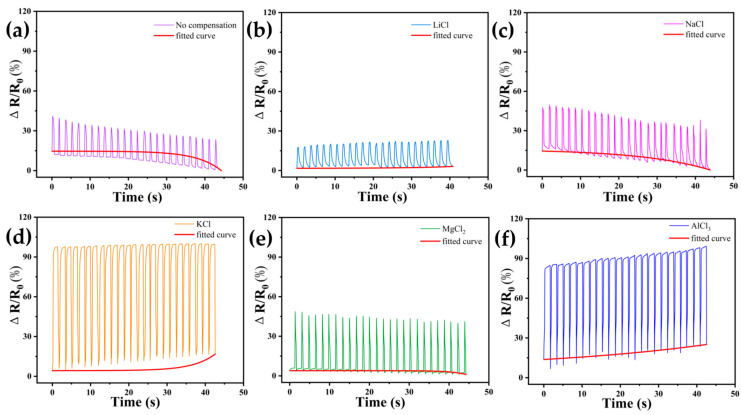
Pressure testing of DN-SPEZ hydrogel in seawater and corresponding stability fitting curves: (**a**) uncompensated experimental; (**b**) LiCl compensation; (**c**) NaCl compensation; (**d**) KCl compensation; (**e**) MgCl_2_ compensation; (**f**) AlCl_3_ compensation.

To further investigate the effectiveness of the DN-SPEZ hydrogel after performing ion compensation in underwater environments, we chose artificial seawater [74] as the study object to evaluate the feasibility of the self-calibrated compensation strategy for hydrogel. As shown in Figure 12a–f, compared with the test results in air (see Figure 9 and Figure 10), the ∆*R/R*_0_ of all the test samples showed an enhancement. And the average deviations calculated for the uncompensated group, LiCl, NaCl, KCl, MgCl_2_, and AlCl_3_ when applied in a seawater environment are 0.01451029, 0.00140914, 0.01439897, 0.01250539, 0.0029094, and 0.01134193, respectively. For the resistive drifts for the uncompensated group in seawater, the average deviation is 10.287, 1.008, 1.161, 4.986, and 1.281 times that of the LiCl, NaCl, KCl, MgCl_2_, and AlCl_3_ compensated groups, respectively. This indicates that in terms of stability during underwater application, the DN-SPEZ hydrogels that we chose for pre-compensation with LiCl, NaCl, KCl, MgCl_2_, and AlCl_3_ ionic solutions still all showed good stability compared to the uncompensated group. The ranking of the compensation effect in both scenarios was LiCl > MgCl_2_ > AlCl_3_ > KCl > NaCl, showing consistency, which further proves that the hydrogel with the self-calibrated compensation strategy has a wide range of applications in amphibious environments. The results of this study not only provide an important reference for the application of hydrogels in amphibious application scenarios, such as human wearable flexible devices, ocean engineering, and underwater detection, but also provide new ideas and directions for further optimization and application of hydrogel-based pressure sensors.

**Figure 13:**
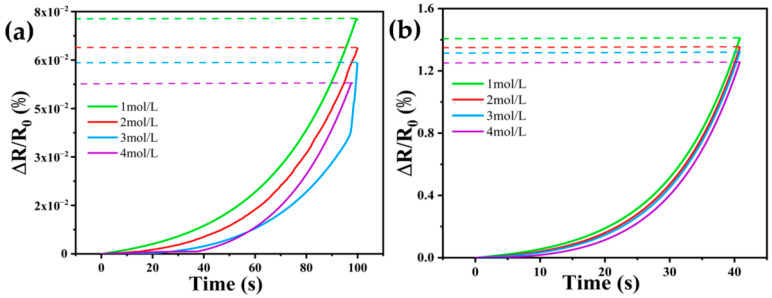
Compensation effect of different concentrations of LiCl compensation solutions: (**a**) compensation in air; (**b**) compensation in seawater.

In order to study the effect of the concentration of the compensation solution on the self-calibration compensation of DN-SPEZ hydrogel, this paper chooses LiCl compensation, which has the best compensation effect in air and sea environments, as an example, and the concentration of the compensation solution is chosen to be 1 mol/L, 2 mol/L, 3 mol/L, and 4 mol/L of LiCl solution in a dosage of 0.05 mL. For the post-compensation pressure response plots of different concentrations of LiCl solutions, the ExpGrow model was selected to fit the floating trend of the response curves with the coordinate origin of the fitted curves, and a comparison of the compensation effect of different concentrations of LiCl in air (Figure 13a) and seawater (Figure 13b) can be observed. When tested in air, the $\bar{\epsilon }$ after LiCl compensation was 0.00007261, 0.00006363, 0.000059, and 0.000052886 for concentrations of 1 mol/L, 2 mol/L, 3 mol/L, and 4 mol/L; and when tested in artificial seawater, the $\bar{\epsilon }$ after LiCl compensation was 0.00140914, 0.001350766, 0.00132311, and 0.001257 for concentrations of 1 mol/L, 2 mol/L, 3 mol/L, and 4 mol/L. L of LiCl after compensation, respectively. A smaller value of $\bar{\epsilon }$ indicates a better compensation effect. The results showed “1 mol/L < 2 mol/L < 3 mol/L < 4 mol/L” for both tests in air and seawater, suggesting that high concentrations of LiCl provide more free carriers when compensating DN-SPEZ hydrogels as the compensating solution penetrates into the hydrogel.

The results of this study not only provide an important reference for the application of hydrogel in amphibious application scenarios, such as human wearable flexible devices, ocean engineering, and underwater detection, but also provide new ideas and directions for the further optimization and application of hydrogel-based pressure sensors.

### 3.6. Multi-Scenario Application of DN-SPEZ Hydrogel Self-Calibrating Compensation Sensor

**Figure 14:**
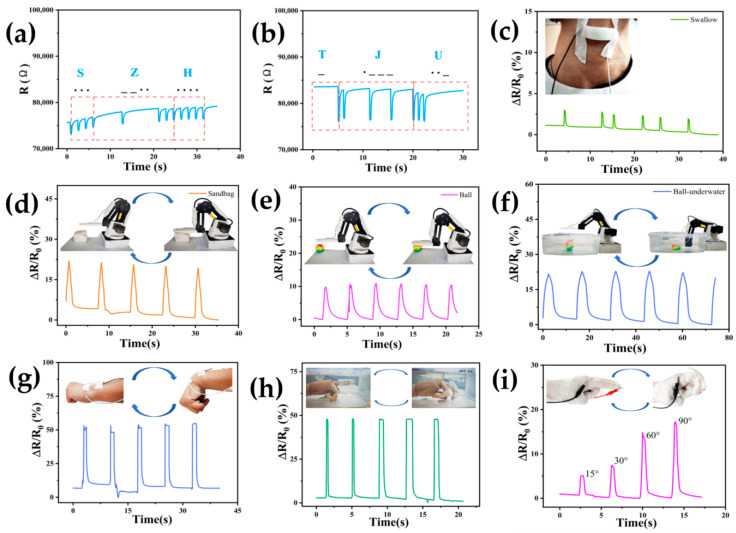
Multi-scenario application testing of DN-SPEZ hydrogel after self-calibration compensation with LiCl. (**a**,**b**) DN-SPEZ hydrogel for Morse code recognition in seawater environments; (**c**) DN-SPEZ hydrogel for monitoring swallowing movements; (**d**,**e**) DN-SPEZ hydrogel attached to robotic arm for sandbag and ball press tests; (**f**) DN-SPEZ hydrogel presses against small balls in seawater; (**g**) DN-SPEZ hydrogel for monitoring wrist flexion; (**h**) DN-SPEZ hydrogel for monitoring finger flexion in seawater; (**i**) DN-SPEZ hydrogel for monitoring finger flexion of different amplitudes.

Based on the self-calibration compensation study, the LiCl-solution-compensated hydrogels showed good stability and pressure-sensitive properties. Figure 14a–i demonstrated the practical application of DN-SPEZ hydrogels in the self-calibration compensated state of LiCl solution (1 mol/L). As shown in Figure 14a,b, the hydrogel was tested in a seawater environment by tapping the hydrogel using Morse code, denoting the name abbreviation “SZH” as well as the school’s name abbreviation “TJU” with a short bandwidth denoting “ . ” and long bandwidth denoting “—”; it can be seen that the hydrogel can still respond effectively to short taps in a seawater environment. Figure 14c demonstrates the application of DN-SPEZ hydrogel as a human swallowing monitoring sensor, specifically for detecting swallowing changes in the pharynx when drinking water. The compensated DN-SPEZ hydrogel sensor can effectively monitor small movements such as swallowing, with feedback ∆*R/R*_0_ reaching 3%. Therefore, the self-calibration compensation strategy is a feasible and effective approach for wearable hydrogel sensors.

When the DN-SPEZ hydrogel in the self-calibrated compensated state of LiCl solution was attached to a 3D-printed hand model, a sandbag press (Figure 14d), a small ball press, and a small ball placed in seawater, the DN-SPEZ self-calibrated sensors fed back the press stresses as shown in Figure 14d–f. Compared to the maximum ∆*R/R*_0_ of 9.8% achieved for the pressing response of the sphere placed in air (Figure 14e), the pressing response of the sphere placed in seawater (Figure 14f) exhibits a larger ∆*R/R*_0_, which can reach a maximum of 21.7%. We believe that the ions in seawater enter the interior of the hydrogel with the osmotic process, which increases the concentration of carriers inside the hydrogel and thus improves the pressure sensing performance, and the pressure from the water is also an important factor contributing to the larger ∆*R/R*_0_.

To demonstrate the sensitivity of the DN-SPEZ self-calibrating hydrogel sensor in human wear scenarios, we encapsulated and fixed it on the wrist using adhesive tape for wrist bending monitoring (Figure 14g). The bending angle ranged from 0° to 60°, and the ∆*R/R*_0_ varied from 0% to 52.1%. Additionally, we enclosed and secured a wooden hand model to monitor fixed-amplitude finger bending (see Figure 14h). The bending angles ranged from 0° to 90°, and the ∆*R/R*_0_ changes ranged from 0% to 47.8%. Finally, we tested the resistance change of the package when bending at different angles on the index finger joint (see Figure 14i). The results showed that the ∆*R/R*_0_ increased as the bending amplitude of the finger increased: 5.2% for bending up to 15°, 7.4% for bending up to 30°, 14.8% for bending up to 60°, and 17.2% for bending up to 90°. The monitoring results demonstrate the feasibility of the DN-SPEZ self-calibrating hydrogel sensor in various wearable scenarios.

## 4. Discussion

In summary, with the application of the self-calibration compensation scheme, DN-SPEZ dual-network hydrogels can be used as strain and pressure sensors. At the same time, the introduction of EG, on the one hand, makes the DN-SPEZ dual-network hydrogel sensors in underwater application scenarios maintain a stable size and shape; they will not be affected by the water and over-expansion, to a certain extent, preventing the hydrogel sensor from undergoing sensing distortion or damage. On the other hand, the DN-SPEZ dual-network hydrogel has better temperature-sensitive characteristics and can still work normally in a −20 °C low-temperature environment, possessing the potential to be used as a sensor in extreme environments.

**Figure 15:**
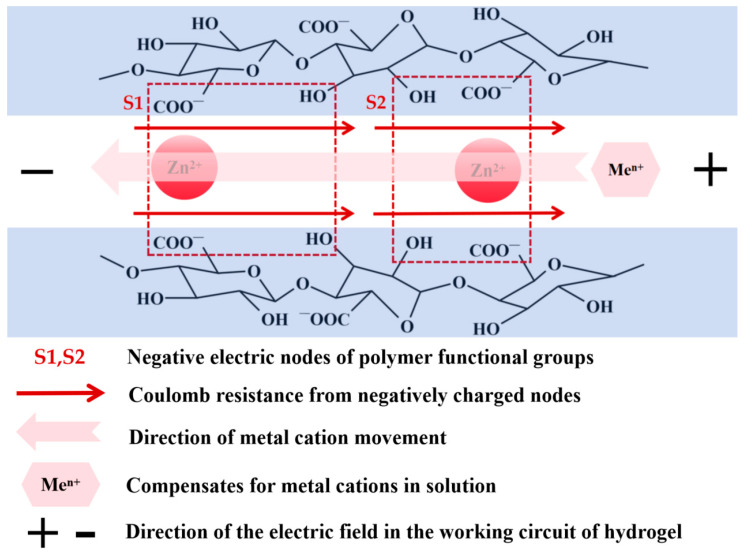
Modeling the polar migration of metal cations within hydrogels in compensating solutions—an example of network structures formed in sodium alginate with Zn^2+^.

Moreover, when the self-calibration compensation program DN-SPEZ dual-network hydrogel is applied to strain and pressure sensors, compared with sensors without self-calibration compensation, the stability of fatigue test and the pressure and strain sensing characteristics of LiCl, NaCl, KCl, MgCl_2_, and AlCl_3_ solutions, for example, are improved to different degrees, reducing the resistance drift phenomenon caused by the hydrogel sensors in the working circuit. The negative impact of the resistance drift phenomenon in the working circuit of the hydrogel sensor is reduced. The results of the subsequent tests in the seawater environment show that although the carriers in the seawater environment enter the hydrogel to improve the pressure sensing performance of the sensor, the compensation effect is consistent with that in the air, which still reflects the advantage of implementing the self-calibration compensation strategy. Further exploring the advantages and disadvantages of the compensation effects of LiCl, NaCl, KCl, MgCl_2_, and AlCl_3_, in addition to the concentration of the compensation solution, we believe that the size of the cation radius is an important factor in determining the compensation effect, but the mobility of the ions in the hydrogel is also affected by the charge carried by the cations, in which the ordering of the compensation effects of ‘LiCl > MgCl_2_ > AlCl_3_’ is exactly the same as that of the charge carried by their corresponding cations. We believe that the ions in the compensating solution migrate polarly under the influence of the electric field of the hydrogel energizing circuit, but the negative nodes (hydroxyl and carboxyl groups) of the polymer functional groups in the structure of the DN-SPEZ hydrogel produce a “Coulombic resistance” to the movement of the positively charged ions, and therefore, although Al^3+^ and Mg^2+^ have higher charge density in the hydrogel, the “Coulomb resistance” of the negatively charged nodes is stronger, and the polar mobility becomes lower accordingly, which also explains the ordering of the compensation effect of ‘LiCl > MgCl_2_ > AlCl_3_’, as shown in Figure 15.

Finally, we also show the multi-scenario test results obtained when the self-calibration compensation scheme was applied to DN-SPEZ dual-network hydrogels to realize applications such as sending messages in Morse code in seawater environments, monitoring swallowing motions, sensing the force of a robotic hand pressing an object in air and an object in water, sensing wrist bending, and sensing underwater bending of fingers.

## 5. Conclusions

The application of the self-calibration compensation scheme in DN-SPEZ dual-network hydrogels provides an ideal and feasible strategy for designing strain and pressure sensors suitable for various amphibious scenarios. Meanwhile, the results of this study also demonstrate that the hydrogel self-calibration compensation strategy has great potential and far-reaching significance in the field of flexible wearable sensors. In hydrogel sensing and monitoring in different application scenarios, in addition to increasing the concentration of the compensation solution within a limited range, we take the ionic radius and the amount of charge carried by the metal cation as measured and select compensation solutions with different compositions purposely to compensate the resistive drift of hydrogel sensors with different demands, and this strategy can effectively realize the controllable self-calibrated compensation of hydrogel sensors.

## Data Availability

No new data were created for this study.
